# Novel Use of Ivabradine for Persistent Sinus Tachycardia in a Patient on Extracorporeal Life Support With Right Ventricular Dysfunction

**DOI:** 10.7759/cureus.62638

**Published:** 2024-06-18

**Authors:** Kristina Akopyan, Aman Shah, Mindaugas Rackauskas, Cynthia Gries, Amir Emtiazjoo, Biplab K Saha

**Affiliations:** 1 Internal Medicine, University of Florida, Gainesville, USA; 2 Cardiology, University of Florida, Gainesville, USA; 3 Thoracic Surgery, University of Florida, Gainesville, USA; 4 Lung Transplant, University of Florida, Gainesville, USA

**Keywords:** ards (acute respiratory distress syndrome), sinus tachycardia, ivabradine, veno-venous ecmo, right ventricular dysfunction

## Abstract

Persistent sinus tachycardia (pST) has been associated with adverse cardiovascular events in critically ill patients. Pharmacological control of heart rate with negative inotropic agents has proven to be safe but could be potentially dangerous in patients with concomitant right ventricular (RV) dysfunction. Ivabradine, a medication devoid of negative inotropy, could be a potentially safe solution for this patient population when adequate heart rate control is desired. A 17-year-old male with a history of vaping developed acute respiratory distress syndrome (ARDS) and RV dysfunction, requiring extra corporal life support (ECLS). He suffered from pST. Given his RV dysfunction, a beta-blocker was avoided, and ivabradine was used safely with improvement of his pST. This case demonstrates the efficacy of ivabradine to reduce heart rate and avoid the use of beta-blockers for patients with RV dysfunction, which could be detrimental. Ivabradine was shown to lower the heart rate without altering hemodynamic parameters.

## Introduction

Sinus tachycardia (ST) is a common finding in critically ill patients in the ICU. A secondary etiology for ST can be identified for majority of these patients, such as hypovolemia, anemia, pulmonary embolism, hyperthyroidism, pain, sepsis, and drug withdrawal [1]. Some patients experience persistent sinus tachycardia (pST) without definite triggers. Whether any intervention should be undertaken to minimize heart rate is debatable. While ST is a normal physiologic response, pST in critically ill patients has been associated with adverse cardiovascular events [2]. The primary concern in treating patients with ST is the precipitation of cardiovascular compromise due to reduced cardiac output. However, strict control of heart rate (HR) with esmolol in patients diagnosed with septic shock has shown improved cardiac output (CO) and systemic vascular resistance (SVR) [3]. Therefore, it is likely that at least in a subset of patients suffering from pST (perceived to be inappropriately high), reduction of the HR may be hemodynamically beneficial. Medications that are commonly used for suppression of the sinus node are beta-blockers and calcium channel blockers; these medications are known to possess negative inotropic properties and could be dangerous, especially in the presence of ventricular dysfunction [4]. Ivabradine specifically blocks the If current, the ionic current that generates the spontaneous diastolic depolarization of the sino-atrial (SA) node. This exerts a negative chronotropic effect on the SA node without affecting ventricular contractility. Ivabradine has been shown to be effective and more tolerable than use of non-selective beta-blockers [5]. We report the case of a patient with acute respiratory distress syndrome (ARDS) and right ventricular (RV) dysfunction on extracorporeal membrane oxygenation (ECMO), where we used ivabradine for pST. To the best of our knowledge, the use of ivabradine in this patient population has not been previously described.

## Case presentation

A 17-year-old male with a history of vaping presented to an outside facility with fever, cough, and sputum production for one week. Initially, he required high-flow nasal cannula oxygen, but his respiratory status rapidly worsened, requiring intubation and mechanical ventilation. He was thought to be suffering from e-cigarette or vaping-associated lung injury (EVALI) and was started on high-dose steroids and broad-spectrum antibiotic therapy. His peak and plateau pressures were high on mechanical ventilation despite using lung-protective ventilatory strategies necessitating venovenous (VV)-ECMO initiation. A single-site dual-lumen crescent cannula (ProtekDuo; LivaNova, London, UK) was placed, and the patient was transferred to our center for further management.

**Figure 1:**
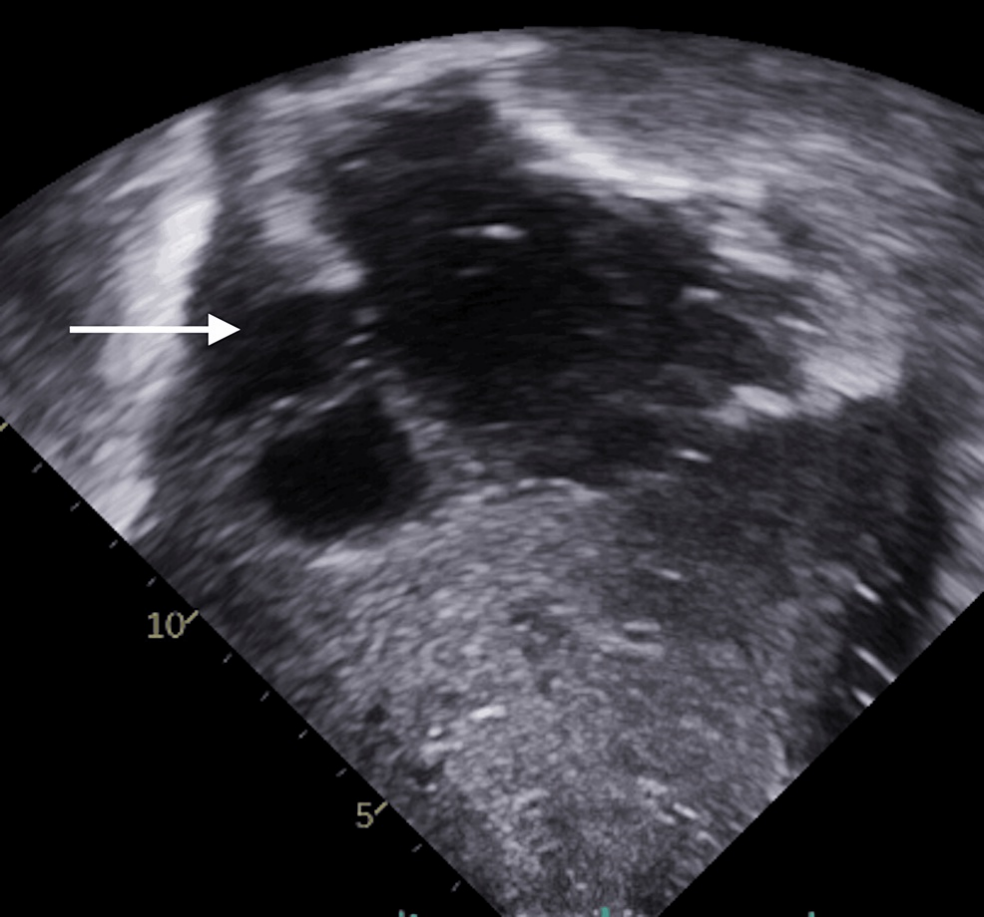
Bedside echo showing dilated right ventricle (RV)

Unfortunately, his hospitalization was complicated by bilateral pneumothoraxes requiring chest tubes, respiratory syncytial virus (RSV) pneumonia, Enterococcus faecalis bacteremia, lung abscess, and candida empyema over the course of two months. He was treated with ribavarin, linezolid, and piperacillin/tazobactam for two weeks and completed a four-week course of micafungin. He also developed a provoked right lower lobe segmental pulmonary embolism, for which he received three months of anti-coagulation. During the ECMO run, he was noted to have RV dysfunction. Transthoracic echocardiogram (TTE) at bedside showed RV dilation but with preserved tricuspid annular plane systolic excursion (TAPSE) of 20 mmHg (Figure 1). The V-V configuration was switched to veno-pulmonary (V-P) configuration to provide RV support and prevent overt RV failure. 

**Figure 2:**
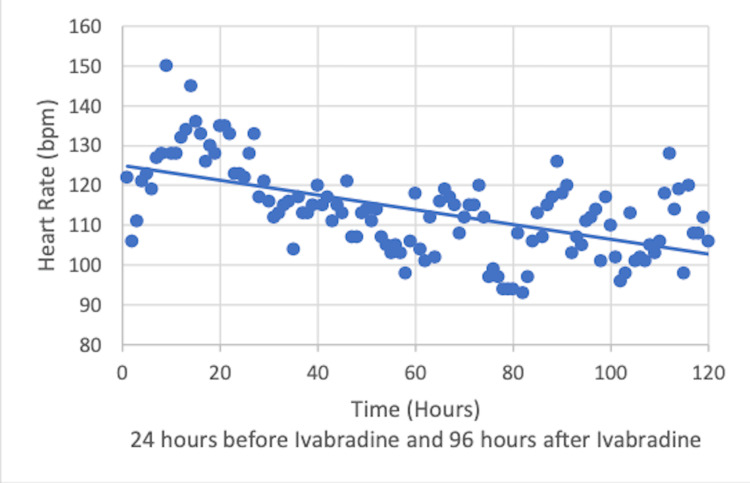
Decline of heart rate after the initiation of ivabradine.

**Figure 3:**
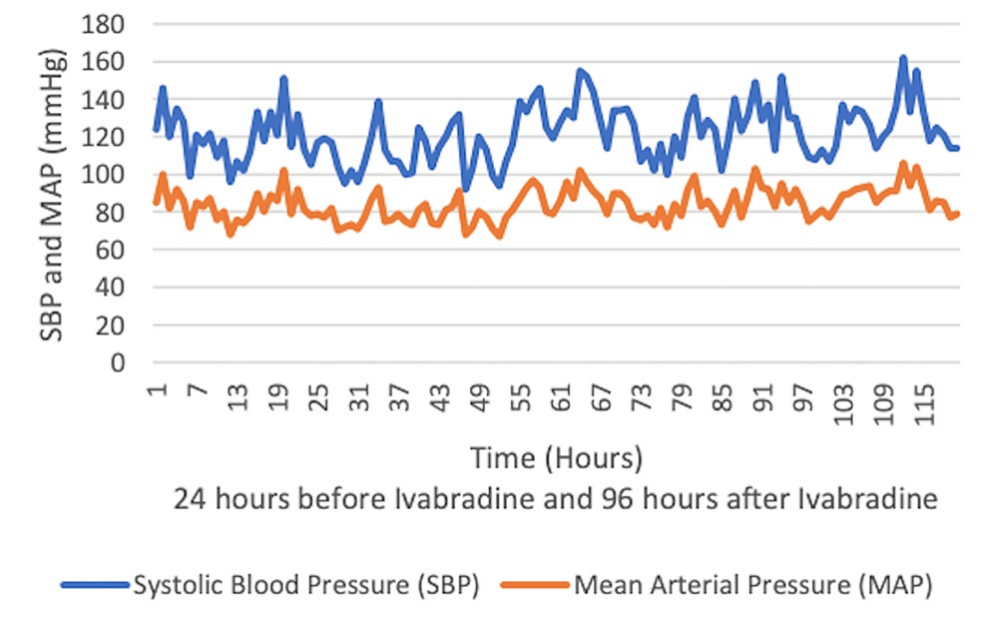
Stable hemodynamic parameters of systolic blood pressure and mean arterial pressure after the initiation of ivabradine.

**Figure 4:**
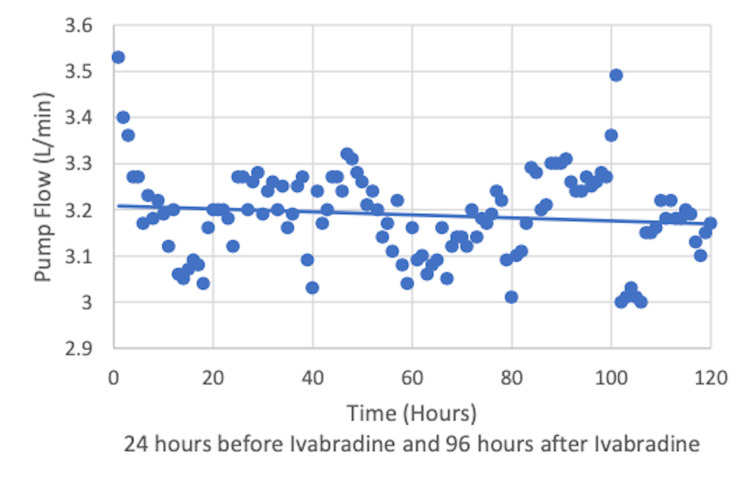
Stable extracorporeal membrane oxygenation (ECMO) pump flow after the initiation of ivabradine

The patient suffered from pST in the range of 120-160 beats per minute (bpm). At the time, he was on appropriate antimicrobials with stable hemoglobin of 9.5 g/dL and receiving treatment for anxiety. Given his RV dysfunction, ivabradine was initiated at 5 mg twice daily under close monitoring of hemodynamics. HR, systolic blood pressure (SBP), mean arterial pressure (MAP), and ECMO pump flow were recorded 24 hours prior to the initiation of ivabradine and followed for 96 hours while on continued ivabradine (Figures 2-4). 

HR declined after ivabradine initiation. The medication was started at 5 mg twice daily and continued for 13 days prior to weaning to 2.5 mg twice daily. It was continued at this dose for 14 days prior to discontinuation due to bradycardia. SBP, MAP, and ECMO pump flow remained stable. Lactic acid during this time also remained stable ranging from 0.7 to 1.2 mmol/L. The patient was on ECMO for a total of 56 days. To evaluate the recovery of the RV, the flow through the extracorporeal circuit was gradually reduced after administration of 2,000 units of unfractioned heparin. The flow was reduced to 1 L/min and bedside echocardiogram was used to assess the RV size and TAPSE. The ProtekDuo was removed as there was no alteration in the RV function with reduction of flow through the circuit. He had a tracheostomy which was removed before his discharge home on 2 L/minute of nasal cannula oxygen.

## Discussion

We have reported a case of severe ARDS complicated by RV dysfunction requiring several weeks of extra corporal life support (ECLS). For our patient, the V-V configuration was switched to a V-P configuration utilizing the ProtekDuo cannulas as an oxygenated right ventricular assist device (oxy-RVAD).

One of the most crucial findings in this case is the safety of ivabradine in this patient population of critically ill patients in the ICU with pST. Ivabradine has been shown to be effective and safe for chronic stable angina in the BEAUTIFUL and SIGNIFY studies [6,7]. It has also been approved for use in patients with heart failure with left ventricular ejection fraction (LVEF) of less than or equal to 35%, as shown in the SHIFT study [8]. Ivabradine acts directly on the SA node by inhibiting the highly selective If current, which leads to a dose-dependent reduction of heart rate without a significant effect on the atrio-ventricular conduction, left ventricular contraction, or blood pressure [9]. Given that ivabradine is an agent limited to the negative chronotropic effect on the SA node without affecting ventricular contractility, we observed improved HR with stable SBP, MAP, and ECMO pump flow. Lactic acid also remained stable, suggesting adequate peripheral oxygen delivery. Animal models with sepsis have been treated with ivabradine to assess the hemodynamic effects. This investigation demonstrated HR control with preservation of blood pressure, CO, and left ventricular systolic function after the use of ivabradine when compared to atenolol [10]. Our patient had sepsis as well as ARDS. The use of ivabradine in patients with sepsis and ARDS needs further evaluation.

The second point is the use of ivabradine in patients with RV dysfunction. pST is often an early physiologic response in patients suffering from RV dysfunction. RV failure in patients with ARDS occurs from a combination of excessive afterload, excessive preload, and insufficient myocardial contractility. Acute pulmonary hypertension (PH) will lead to an increased RV afterload and reduce RV contractility. This will then lead to RV dysfunction, followed by tachycardia and increased stroke work, resulting in increased oxygen demand [11]. However, in the setting of echocardiographically stable RV and worsening ST, the ST can have detrimental effects. It could reduce the RV preload, eventually affecting the left ventricular stroke volume. The International Society for Heart and Lung Transplantation (ISHLT) consensus statement recommends against routine use of beta blockers to prevent ST in RV failure [12]. Rate-lowering drugs such as beta blockers lengthen the diastolic period, which increases the SV and CO in systolic left ventricular failure, but they are not effective and can even be detrimental in heart failure with preserved ejection fraction [13]. Beta-blockers and calcium channel blockers have a negative ionotropic effect, and they may hamper RV contractility and promote cardiac decompensation [14]. In our patient with RV dysfunction and supra-normal CO, we did not observe any adverse effects with the use of ivabradine for pST. pST in patients with PH and RV failure has been associated with RV remodeling and fibrosis, which could potentially be prevented by adequate control of the heart rate [15].

## Conclusions

Our patient suffered from ARDS secondary to EVALI requiring VV-ECMO, which was further complicated by bilateral pneumothoraxes with bronchopleural fistula. He developed RV dysfunction with echocardiographically stable RV function after initiation of V-P ECMO. For this patient, using ivabradine proved beneficial in controlling HR and maintaining his hemodynamics. This case highlights the need for further research into the treatment of pST with ivabradine to avoid potential adverse effects of beta-blocker use, especially in the setting of RV dysfunction.
